# Ethnicity and equity of access to a tier 4 national tic service

**DOI:** 10.1192/bjb.2025.10166

**Published:** 2026-06

**Authors:** Saam Idelji-Tehrani, Nimmi Parikh, Matteo Catanzano, Isabel Archer, Madiha Shoaib, Holan Liang

**Affiliations:** 1 Royal Alexandra Children’s Hospital, Brighton, UK; 2 Sussex Partnership Foundation Trust, Worthing, UK; 3 Brighton and Sussex Medical School, Brighton, UK; 4 The Tic Service, Great Ormond Street Hospital for Children, London, UK; 5 Department of Child & Adolescent Psychiatry, King’s College London, London, UK; 6 South London and Maudsley NHS Foundation Trust, London, UK; 7 Autism Research Centre, University of Cambridge, Cambridge, UK; 8 Psychological and Mental Health Services, Great Ormond Street Hospital for Children, London, UK

**Keywords:** Child and adolescent psychiatry, epidemiology, quality improvement, stigma and discrimination, transcultural psychiatry

## Abstract

**Aims and method:**

To examine whether unconscious and systemic biases regarding ethnicity have an impact on equity of access to a national tic service for children and young people (CYP) at Great Ormond Street Hospital for Children, London, UK. We retrospectively reviewed triaged referrals over an 18-month period and examined differences in triage decision, re-referrals required before acceptance and symptom severity at initial assessment by clinician-perceived and self-assigned ethnicity.

**Results:**

There was no evidence of an unconscious bias within the triage process. CYP from racially minoritised ethnic backgrounds were underrepresented and presented with greater overall need at initial assessment.

**Clinical implications:**

Better recording of ethnicity is a requisite starting point for research. We encourage local services to audit ethnicity of the CYP they refer to national and specialist services. Findings call for greater awareness of challenges faced by patients from racially minoritised ethnic backgrounds.

People from minoritised ethnic backgrounds are often underrepresented in mental health services.^1^ De la Cruz et al^2^ illustrated that minoritised ethnic groups with obsessive–compulsive disorder were severely underrepresented in secondary and tertiary mental health services in a large UK mental healthcare trust. Studies^3,4^ have demonstrated that, compared with White British children and young people (CYP), CYP of minoritised ethnic backgrounds are less likely to access mental health services through voluntary care pathways and more often through referrals from social care or the criminal justice system.

Sharland et al^5^ reported that people born outside of the UK and from Asian ethnic backgrounds were likely to be underrepresented in Improving Access to Psychological Therapies (IAPT) services nationally. Crucially, self-referral rates of minoritised ethnic groups to an IAPT service were found to be lower than in White British groups.^6^ Strikingly, as part of the same study, all minoritised ethnic groups were less likely to receive an assessment compared with the White British group, and of those who were assessed, minoritised ethnic groups were less likely to be treated. Farooq et al^7^ additionally found that CYP from minoritised ethnic backgrounds presenting in crisis to hospitals (with self-harm) were less likely to be offered a psychosocial assessment than White CYP. In forensic child and adolescent mental health services (CAMHS), it has been found that Black and dual heritage CYP are disproportionately disadvantaged, being less likely to be diagnosed with a neurodevelopmental disorder compared with White CYP.^8^

Ethnic inequalities in access to healthcare are, however, not unique to mental healthcare. Inequity has been found within cardiology^9^ and urology^10^ services. Teager et al^11^ also found that minoritised ethnic groups in the UK were underrepresented within a neuropsychology service relative to regional population prevalence. People from minoritised ethnic groups furthermore have been shown to have poorer outcomes and healthcare experiences in general.^12^

Unconscious biases around ethnicity can influence decisions pertaining to access to healthcare. Miu et al^13^ demonstrated that patients perceived as belonging to a minoritised ethnic group – referred to in the study as ‘Black, Asian and minority (non-White) ethnic (BAME)’ – were more likely to be discharged from an ear, nose and throat (ENT) service following a missed appointment compared with those perceived to be White British.

## The rationale for the present study

The Tic Service at London’s Great Ormond Street Hospital for Children (GOSH) is commissioned to offer comprehensive assessment and treatment for CYP with tics, Tourette syndrome and tic-like behaviours. It is a national service, meaning that it is a centre of expertise, and referrals are required to come from community paediatric or mental health services rather than general practitioners. The prevalence of Tourette syndrome is estimated to be 1% in CYP across most cultures across the world, although it may be seen less frequently in some cultures.^14–16^

Anecdotally, within the GOSH Tic Service, it was felt that minoritised ethnic groups were underrepresented. Therefore, with this study, we aimed to establish whether there may be an unconscious bias within the referral triage process of the Tic Service, and whether there may be systemic barriers that have an impact on referrals and acceptance to the service.

The specific objectives of the study were to determine the following.

- What proportion of CYP referred are from White versus racially minoritised ethnic backgrounds?
- Is there a difference in the acceptance/rejection rate for referrals of CYP who are from racially minoritised ethnic backgrounds?
- Are CYP from racially minoritised ethnic backgrounds more likely to require multiple referrals to the service prior to acceptance compared with CYP from White ethnic backgrounds?
- Of those accepted for assessment, are there differences in symptom severity between CYP who are from White versus racially minoritised ethnic backgrounds?

Pertinently, we must acknowledge the use of language in this paper. Unless specified, we have chosen to use the terms ‘minoritised ethnic group’ and ‘racially minoritised ethnic group’. When discussing our own results, we predominantly utilise the term racially minoritised ethnic group to reflect the binary subdivision utilised (‘White’ versus ‘Non-White’).

## Method

We retrospectively analysed two data-sets of patients who were referred to the Tic Service at GOSH between May 2021 and November 2022. We have termed these the self-assigned and the perceived ethnicity data-sets. This project was registered with and approved by the hospital’s clinical governance and safety department as an audit project (ID: 3471) and formal ethical approval was therefore not required. Patient consent was not required as members of the research team were part of the clinical team.

### Perceived ethnicity data-set

A referral data-set, consisting of the names of all patients referred to the Tic Service over the study period, was collated by staff from the Tic Service and analysed to explore questions around unconscious biases. In the real-life setting, when deciding whether to accept or decline a referral at the point of triage, patient ethnicity is not available to the clinician. Hence, it may be assumed based solely on the patient’s name. This data-set included information relating to name, patient ID, referral date, referral outcome and reason for rejection if a case was rejected. CYP were declined for assessment if one or more of the following four criteria were not met: (a) CYP must be referred by their local CAMHS or their community paediatrician and they must be open to that service; (b) CYP must be presenting with tics in the moderate-to-severe range; (c) local CAMHS must be the referrer if the young person has significant co-occurring mental health difficulties; (d) and the CYP’s general practitioner must be informed of the referral to the service.

Our method to assess for unconscious name bias is based on the procedure described in Miu et al’s^13^ study, where they examined the association between perceived ethnicity rates and discharge from an ENT service. We gave five (White or minoritised ethnic) assessors a list of patient names as they appeared on patient records. Each assessor was asked to independently assign whether they perceived the patient’s name to be from a ‘White’ or ‘Non-White’ (i.e. racially minoritised) ethnic background. Each assessor was masked as to whether the patient was offered an assessment. The modal ethnicity was then taken as the ‘perceived ethnicity’.

To determine a degree of certainty with the perceived ethnicity gradings, it was agreed that CYP with an interrater ethnicity consensus rating ≥80% (i.e. four out of five assessors agreeing) would be included in the data analysis. We also performed Cohen’s kappa statistical test for interrater reliability between perceived ethnicity and self-assigned ethnicity, to further validate and assess the accuracy of our method.

To examine the possible impact of unconscious bias on acceptance, we retrospectively compared acceptance rates between the perceived ethnicity groups. A *P*-value less than 0.05 was considered significant and analysis was performed using the chi-squared test. With 363 participants and *α* = 0.05, we were sufficiently powered (0.80) to detect a small effect size (Cohen’s ω) of 0.14 for the chi-squared analysis.

### Self-assigned ethnicity data-set

Data regarding self-assigned ethnicity, gender and clinician-rated symptom severity were extracted from electronic medical records. These data were then utilised to explore rate of acceptance and symptom severity by self-assigned ethnicity. The data-set was recoded as a binary variable (‘White’ versus ‘Non-White’). Binary variables were used because of the small numbers found in individual groups, which also prevented analysis of a greater number of discrete minoritised ethnic groups.

Symptom severity is recorded on the electronic medical records system by clinicians following an initial assessment using two well-validated clinical rating scales – the Yale Global Tic Severity Scale (YGTSS)^17^ and the Child Global Assessment Scale (CGAS).^18^ For the YGTSS, the Total Yale Global Tic Severity Score is used.^17^ We performed Mann–Whitney *U*-tests to determine whether there was an association between binary ethnicity and symptom severity.

The self-assigned ethnicity data-set is smaller than the perceived ethnicity data-set as ethnicity data were missing for many CYP. Acceptance to the service presents clinicians an opportunity to record ethnicity. No additional opportunities to code for ethnicity were available if the individual was rejected at the point of referral, and therefore there was a higher rate of missing ethnicity data for CYP rejected from the service. CYP without ethnicity data or YGTSS/CGAS scores were not included in the respective analyses.

## Results

Cohen’s kappa statistical test revealed substantial agreement (κ = 0.62; *P* ≤ 0.001) between the perceived ethnicity and self-assigned ethnicity.

### Perceived ethnicity data-set

The Tic Service received 421 total referrals during the 18-month time frame. Once perceived ethnicity was coded, 395 total referrals met a rating consensus of ≥80%. From those 395 referrals, 32 repeat referrals were removed, leaving a total of 363 individual referrals meeting consensus threshold. The majority of CYP within the data-set were perceived as White (*n* = 329; 91%).

There was no significant association between perceived ethnicity and rate of acceptance to the Tic Service; 128 CYP (39%) who were perceived to be White were accepted, compared with 8 (24%) perceived as Non-White (*χ* ^2^ = 3.1097; *P* = 0.078), although proportional acceptance figures were higher for the perceived White subgroup.

Table 1 descriptively demonstrates total referrals (*n* = 395), including re-referrals to capture reasons for rejection. Examination of re-referrals and acceptance rates revealed no statistically significant association between rate of re-referrals and perceived ethnicity, as assessed by Fisher’s exact test (*P* = 1.00). Similarly, no statistically significant association, as assessed by Fisher’s exact test (*P* = 1.00), was found between the rate of acceptance on re-referral and perceived ethnicity. In total, 19/25 CYP (76%) perceived to be White and 3/3 CYP (100%) perceived to be Non-White were accepted on re-referral.

**Table 1:** Frequency of acceptance and rejection (including reasons for rejection) of young people at triage by binary perceived ethnicity, using Tic Service data (*n* = 395)

Acceptance/rejection	Perceived ethnicity, *n* (%)	
	White	Non-White
Accepted	145 (41)	11 (30)
Reason for rejection		
Needs community CAMHS	137 (38)	16 (43)
Insufficient information	16 (4)	3 (8)
Inappropriate referrer	29 (8)	2 (5)
Inappropriate referral	21 (6)	4 (11)
Age	3 (1)	0 (0)
Missing data	7 (2)	1 (3)

CAMHS, child and adolescent mental health services.

### Self-assigned ethnicity data-set

Utilising the self-assigned ethnicity data-set, a total of 369 referrals were received by the Tic Service. Of these, 108 were coded with a self-assigned ethnicity. Once repeat referrals were removed, a total of 93 individual CYP had been referred to the Tic Service with a self-assigned ethnicity coded (Table 2). The majority of patients with a self-assigned ethnicity were White British (*n* = 72; 77%).

**Table 2:** Frequency of self-assigned ethnicities as coded in patients’ electronic medical records (*n* = 93)

Self-assigned ethnicity	Frequency, *n* (%)
Asian Bangladeshi	1 (1)
Asian Indian	2 (2)
Asian Pakistani	1 (1)
Black African	1 (1)
Black Caribbean	1 (1)
Mixed White and Black African	1 (1)
Mixed White and Black Caribbean	1 (1)
Other Black background	1 (1)
Other ethnic group	3 (3)
Other mixed background	2 (2)
Other White background	5 (5)
White British	72 (77)
White Irish	2 (2)

When self-assigned ethnicities were recoded as a binary variable, 79 CYP (85%) were from White ethnic backgrounds, and 14 (15%) were from Non-White ethnic backgrounds. No statistically significant association was found between the rate of acceptance and self-assigned ethnicity, as assessed by Fisher’s exact test (57% White CYP versus 57% Non-White CYP; *P* = 1.00).

Table 3 descriptively shows total referrals (*n* = 108), including re-referrals, to capture reasons for rejection. Re-referrals were once again explored. At the point of re-referral 8 out of 79 White CYP (10%) and 2 out of 14 Non-White CYP (14%) were accepted. There was no statistically significant evidence of any association between self-assigned ethnicity and rates of acceptance on re-referral (*P* = 0.643).

**Table 3:** Frequency of acceptance and rejection (including reasons for rejection) of young people at triage by binary self-assigned ethnicity, using data from patients’ electronic medical records (*n* = 108)

Acceptance/rejection	Binary self-assigned ethnicity, *n* (%)	
	White	Non-White
Accepted	46 (52)	10 (53)
Reason for Rejection		
Needs community CAMHS	25 (28)	1 (5)
Insufficient information	5 (6)	2 (11)
Inappropriate referrer	6 (7)	3 (16)
Inappropriate referral	4 (4)	3 (16)
Age	1 (1)	0 (0)
Missing data	2 (2)	0 (0)

CAMHS, child and adolescent mental health services.

An association was found between CGAS scores at initial assessment and self-assigned binary ethnicity. CGAS median scores were seven points lower (Table 4) for Non-White CYP compared with White CYP (*z* = 2.175; *P* = 0.027). Lower CGAS scores indicate greater global impairment in functioning. No statistically significant association was found between YGTSS scores and self-assigned binary ethnicity (median difference 4; *z* = −0.493; *P* = 0.637).

**Table 4:** Median scores, interquartile range and sample sizes of outcome measures, using data from patients’ electronic medical records

Binary self-assigned ethnicity	CGAS (*n* = 41)			YGTSS (*n* = 47)		
	Median (IQR)	*n*/*N* total^a^	*P*	Median (IQR)	*n*/*N* total^a^	*P*
White (*n* = 79)	46 (43–51)	36/79	0.027	53 (38–65)	41/79	0.637
Non-White (*n* = 14)	39 (38–40)	5/14		49 (46–61)	6/14	

CGAS, Child Global Assessment Scale; YGTSS, Yale Global Tic Severity Scale.

a. *n*/*N* total demonstrates the number of children and young people (CYP) with a CGAS/YGTSS score against the total number of CYP within each binary self-assigned ethnicity category.

## Discussion

### What proportion of CYP referred are from White versus racially minoritised ethnic backgrounds?

Within the self-assigned ethnicity data-set, 77% of patients seen by the Tic Service were White British, with another 8% identifying as coming from other White groups (85% White). By contrast, 91% of referrals in the perceived ethnicity data-set were coded as White. There appears to be an overrepresentation of White/White British populations seen within the Tic Service, particularly when compared with data from the Office for National Statistics (ONS) for ethnicity in London. Across England, the percentage of the population who identified a belonging to White ethnic groups in 2021 was 81%.^19^ Within London, the percentage population who identified as belonging to White British and White Other groups was 53.8%, whereas in South East England it was 86.3%.^19^ These figures, and their comparison with the self-assigned ethnicity data-set, appear to reflect much of the previous research highlighting inequalities in access to mental health services, in that White populations appear to have slightly enhanced access. Moreover, given that the Tic Service is based in London and receives many of its referrals from London and the Greater London area, one would expect a higher level of referrals from racially minoritised ethnic groups, which is not presently corroborated by our findings.

### Is there a difference in the acceptance/rejection rate for referrals of CYP from racially minoritised ethnic backgrounds?

As regards the presence of unconscious bias in the referral triage process, this was found to be non-significant using self-assigned and perceived ethnicity data. Once re-referrals were examined, there was no evidence to suggest that CYP from Non-White ethnic backgrounds were more likely to be rejected than CYP from White ethnic backgrounds at the point of re-referral. Although it is positive to find that there was no significant unconscious bias in this service, it could be considered as unsurprising, given that the service was willing to put itself under scrutiny in this way.

### Are CYP from racially minoritised ethnic backgrounds more likely to require multiple referrals prior to acceptance compared with those from White ethnic backgrounds?

Despite rates of re-referral to the Tic Service by ethnicity being statistically non-significant, it is interesting to note that within the larger perceived ethnicity data-set, 43% of CYP rejected on initial referral were rejected owing to the lack of involvement of community CAMHS, considered to be needed in the care of a child with mental health needs. In practical terms, our findings may hint towards specific barriers for CYP from Non-White backgrounds in accessing national and specialist services. In particular, the necessity for a local CAMHS team’s involvement may in fact pose a significant barrier to racially minoritised ethnic groups. Lack of awareness of mental health services, cultural stigma and reluctance to utilise mental health services have all been identified as significant barriers to accessing CAMHS.^4,20^ Difficulties with accessing services may lead to delays, which can therefore lead to an increase in symptom severity if not treated over time.^4^ Furthermore, if CYP from minoritised ethnic backgrounds are less likely to be assessed and/or treated,^6^ then it is likely they will need to present to services with greater symptom severity in order to be referred, offered an assessment and offered treatment.

### Of those accepted for assessment, are there differences in symptom severity between CYP from White versus racially minoritised ethnic backgrounds?

Our findings also suggest that CYP from Non-White ethnic backgrounds may have greater overall need at the point of assessment by the Tic Service. This is not reflected in tic severity but in global presentation, which may imply unmet need in comorbid mental health conditions. However, owing to missing data, possible confounds not accounted for and small sample sizes in the Non-White group (*n* = 5 for CGAS and *n* = 6 for YGTSS), results should be interpreted with adequate caution.

### Study limitations

There are several limitations to this study. First, the study utilises a novel method of identifying ethnicity, which has not been widely validated. We can, however, suggest that the method used to estimate ethnicity appears to be reliable, as shown by the degree of corroboration between the self-assigned ethnicity and the consensus perceived ethnicity. We invite replication and validation of this methodology, not merely in this particular clinic but also in other specialist clinics.

Second, the self-assigned ethnicity data-set was limited by a high proportion of CYP and their families not disclosing their ethnicity or selecting ‘Prefer not to say’. Conclusions from the statistical analyses performed must therefore be tempered because of small sample sizes. This limitation is, however, not unique to this study. Ethnicity information has been found routinely to be subject to missing data, affecting reliability.^12,21^ To be able to fully understand whether there is an ethnicity bias, more effort will be required to capture high-quality ethnicity data.

Furthermore, it must be acknowledged that although the Tic Service is a national service based in London, it will most likely receive most of its referrals from London and Greater London area, and thus the population base for referrals is unclear.

Although the distinction between White and Non-White ethnic groups in this study helps to broadly document inequalities faced by racially minoritised ethnic groups, aggregate ethnic categories lack nuance and do not capture the burden faced by specific minoritised ethnic groups (both in the White and Non-White groups).^21,22^ For example, the distinction does not capture the impact on other minoritised ethnic groups who are White presenting, such as Irish traveller and Jewish communities, and similarly the experiences of mixed-race populations. Nor do they consider intersectionality with other areas of difference that bring about inequity, such as socioeconomic status. Future research, with larger data-sets, should strive to explore the burdens faced by specific minoritised ethnic groups rather than aggregate categories.

Finally, it is pertinent to note that this study can only identify barriers in potential referral bias for those who seek help or come in contact with the referral pathway, and thus there may be barriers to access that cannot be picked up through this study. Bansal et al^1^ highlight that experiences of racism, migration, religion and complex trauma may be more relevant than crude ethnic groupings in understanding inequalities and their persistence within mental healthcare. Stigma and fear of gossip, for example, have been found through qualitative research to be crucial barriers to CAMHS utilisation by South Asian families in Scotland.^23^ Future qualitative research with CYP and their families, exploring their experiences accessing national and specialist services with any associated individual and structural obstacles, will be crucial in understanding inequalities and building representative and culturally nuanced services for children’s mental health.

### Clinical and research implications

There needs to be a greater awareness of the challenges faced by patients from minoritised ethnic backgrounds in accessing mental health services in a timely manner. Better recording of ethnicity is a requisite starting point for any future research and further research should focus on the systemic barriers faced by minoritised groups in accessing national and specialist services. The Tic Service is just one of many national and specialist services and we would recommend that all services audit their triage decisions based on ethnicity to ensure fair access. More importantly, as our findings indicate discrepancies between base population ethnicity and ethnicity of CYP referred, we would encourage local services to audit ethnicity of CYP they refer on to national and specialist services to ensure equity of access to national expertise.

## Data Availability

The data that support the findings of this study are available on request from the corresponding author. The data are not publicly available owing to restrictions, e.g. containing information that could compromise the privacy of research participants.

## About the authors

**Saam Idelji-Tehrani** is a consultant child and adolescent psychiatrist at the Royal Alexandra Children’s Hospital, Brighton, UK. **Nimmi Parikh** is a senior clinical psychologist in the Tic Service, Great Ormond Street Hospital for Children, London, UK. **Matteo Catanzano** is an Academic Clinical Fellow in Child and Adolescent Psychiatry at King’s College London, London, UK, and a Core Psychiatry Trainee with South London and Maudsley NHS Foundation Trust, London, UK. **Isabel Archer** is an Undergraduate Psychology Placement Student in the Tic Service, Great Ormond Street Hospital for Children, London, UK. **Madiha Shoaib** is an Undergraduate Psychology Placement Student in the Tic Service, Great Ormond Street Hospital for Children, London, UK. **Holan Liang** is a Senior Clinical Research Associate in the Autism Research Centre, University of Cambridge, Cambridge, UK, and an honorary consultant child and adolescent psychiatrist with the Psychological and Mental Health Services, Great Ormond Street Hospital for Children, London, UK.
